# Evidence of restorative therapies in the treatment of Peyronie disease: A narrative review

**DOI:** 10.1590/S1677-5538.IBJU.2024.9920

**Published:** 2024-08-10

**Authors:** Francesco Costantini Mesquita, Rodrigo Barros, Thiago Fernandes Negris Lima, David Velasquez, Luciano A. Favorito, Edoardo Pozzi, James Dornbush, David Miller, Francis Petrella, Ranjith Ramasamy

**Affiliations:** 1 University of Miami Miller School of Medicine Desai Sethi Urology Institute Miami FL USA Desai Sethi Urology Institute, University of Miami Miller School of Medicine, Miami, FL, USA; 2 Universidade Federal Fluminense Hospital Universitário Antônio Pedro Niterói RJ Brasil Serviço de Urologia, Hospital Universitário Antônio Pedro - Universidade Federal Fluminense - UFF, Niterói, RJ, Brasil; 3 Hospital Memorial Arthur Ramos Maceió AL Brasil Serviço de Urologia, Hospital Memorial Arthur Ramos, Maceió, AL, Brasil; 4 Universidade Estadual do Rio de Janeiro Unidade de Pesquisa Urogenital Rio de Janeiro RJ Brasil Unidade de Pesquisa Urogenital, Universidade Estadual do Rio de Janeiro – UERJ, Rio de Janeiro, RJ, Brasil; 5 AU/UGA Medical Partnership Athens GA USA AU/UGA Medical Partnership, Athens, GA, USA

**Keywords:** Penile Induration, Platelet-Rich Plasma, Extracorporeal Shockwave Therapy

## Abstract

**Objective:**

To describe the evidence of Platelet Rich Plasma (PRP), Stem cells therapy (SCT) and Extracorporeal shockwave therapy (ESWL) for the treatment of Peyronies disease (PD), including information from the main urological society guidelines.

**Materials and Methods:**

A literature review of PubMed articles published between 2000 and 2023 was conducted, utilizing keywords such as "Peyronie's Disease", "Penile curvature", "Platelet Rich Plasma", "Stem cells", and "Extracorporeal shockwave therapy". Only full-text articles in English were included, excluding case reports and opinions.

**Results:**

A considerable number of clinical trials were conducted using PRP penile injections for therapy of PD, showing reduction of curvature, plaque size and improvement in quality of life. Preclinical studies in rats have shown the potential benefit of adipose-derived stem cells, with improvements in erectile function and fibrosis. Human studies with mesenchymal stem cells demonstrated promising results, with reduction of curvature and plaque size. ESWL effects on PD were investigated in randomized clinical trials and demonstrated no significant impact in curvature or plaque size, but reasonable effect on pain control.

**Conclusion:**

Restorative therapies has emerged as an innovative treatment option for PD and the results from current studies appear to be promising and demonstrated good safety profile. Unfortunately, due to scarce evidence, PRP and SCT are still considered experimental by American Urological Association (AUA) and European Association of Urology (EAU) guidelines. ESWT is recommended, by the same guidelines, for pain control only. More high-quality studies with long-term follow-up outcomes are needed to evaluate efficacy and reproducibility of those therapies.

## INTRODUCTION

Peyronie's disease (PD) is a disorder of the penis resulting in pathological curvature which may be associated with painful erections, penile deformity, erectile dysfunction (ED) and impairing of penetrative sexual intercourse (1). Prevalence reported range from 0.4-20%, as higher in patients with prostate cancer or diabetes, in the fifth decade of life (2–4).

The treatment of PD is a challenge. There are many oral options such as potassium para-aminobenzoate (POTABA), pentoxifylline and colchicine, with limited data available and lack of proven benefit. Intralesional injections of Collagenase clostridium histolyticum (CCH) may be offered to patients who desire non-surgical treatment at an earlier stage in the disease process. However, it is a treatment indicated to selected patients, with high costs, diverse experience and the lack of ideal treatment regimen (5).

Penile injections of Platelet Rich Plasma (PRP) and Stem Cell Therapy (SCT), extracorporeal shockwave therapy (ESWT), are potential restorative therapies that gained popularity for the treatment of PD. In recent years, there has been a substantial increase in the number of studies involving the use of these restorative therapies for PD treatment (6–8).

In this review, we describe the evidence of PRP, SCT and ESWL for the treatment of PD, including information from the main urological society guidelines.

## MATERIALS AND METHODS

We analyzed published papers contained in the PubMed between 2000 and 2023 searching by the following key words: "Peyronie's Disease"; "Penile curvature"; "Platelet Rich Plasma"; "Stem cells"; "Extracorporeal shockwave therapy". The literature sources were limited to full-text articles and open-access journals published in English publications. Case reports, editorials and opinions of specialists were excluded.

## RESULTS

This narrative review provides an overview of the current literature involving the evidence of the restorative therapies for the treatment of PD.

### Platelet Rich Plasma (PRP)

Autologous PRP therapy, enriched with growth factors and platelets, is currently being explored for its potential in treating PD. This therapy, while approved by the Food and Drug Administration (FDA) for orthopedic uses, is yet to receive approval for urological conditions (9). The presence of growth factors such as VEGF, PDGF, FGF, and TGF-β in PRP contributes to tissue regeneration by modulating processes like stem cell migration, inflammation, angiogenesis, and wound healing (10). PRP's mechanism of action suggests its potential effectiveness during the acute inflammatory stage of PD. This hypothesis is supported by studies in other fields of restorative medicine, where PRP has shown promise in enhancing wound healing and tissue repair (11). The anti-inflammatory properties of PRP, as evidenced in orthopedic literature, may contribute to its therapeutic effects in PD by reducing plaque-associated inflammation (12).

In terms of clinical application, the technique of PRP preparation and injection protocol is also a critical factor. Variability in PRP preparation methods can lead to differences in the concentration of platelets and growth factors, potentially influencing treatment outcomes (13, 14). Standardization of these protocols is essential for comparing results across studies and for the development of effective treatment guidelines.

The safety profile of PRP in this context appears favorable, with most studies indicating only minor side effects like slight pain, mild penile bruising, ecchymosis, hematomas as well as transient hypotension (15).

However, the efficacy of PRP in Peyronie's disease is less clear. Achraf et al. showed 65 patients with PD, divided into two groups based on the severity of penile curvature, the first with a curvature between 25 and 35° and the second between 35 and 45°. They underwent an average of 6.1 PRP injections each group. Results showed notable curvature reduction in both groups, with an average decrease of 16.8° in the first group and 17.3° in the second group, suggesting PRP's potential as a safe and effective PD treatment (16). Another prospective study evaluated the tolerance and effects of intra-plaque PRP injections in men with PD. After three injections performed 15 days apart in 17 patients, a decrease of 11.8º of penile curvature was observed without any noted side effects (17). Other studies, with different protocols, demonstrated positive results with PRP in improvement in penile curvature and erections, reduction in plaque size and pain, and are depicted in Table-1 (16–24).

**Table 1:** Studies examining the effects of PRP on PD.

STUDY	YEAR	SAMPLE SIZE	STUDY DESIGN	INTERVENTION (PRP)	CONTROL GROUP	OUTCOME MEASURES	KEY FINDINGS	LIMITATIONS
**Virag et al. (** **18** **)**	2017	90 patients	Prospective Cohort Study	8 mL of PRP combined with HA, injected 4 times within 2 months, additional monthly sessions if necessary	None	Penile deformation, TA thickening, presence and size of calcifications, PD and sexual function questionnaires	Significant improvement in angulation and thickening after 4 sessions; 73.3% of patients showed satisfactory improvement; younger patients achieved better results; mean reduction in angle of 16.54°, representing an average reduction of 39.65%	No control group
**Notsek, Boiko M. (** **19** **)**	2019	59 patients	Randomized Controlled Trial	Intralesional PRP injections	Intralesional injections of 0.9% sodium chloride	Curvature angle, plaque size, plaque softness, IIEF-5, pain presence	In the PRP group: 50% angle decrease, 50% plaque size reduction, 59.4% achieved plaque softening, 56.3% enhancement in erectile function (IIEF-5), 84% pain reduction. Control group had significantly lower improvements in these areas.	Longer-term follow-up needed
**Achraf et al. (** **16** **)**	2022	65 patients	Prospective Study	Intralesional PRP injections	None	Curvature angle, erectile function, pain during intercourse	Angulation improved by an average of 16.8° in the first group (25-35° curvature) and 17.27° in the second group (35-45° curvature). Pain during sex decreased significantly; improvement in erectile function	No control group
**Farrag et al. (** **20** **)**	2022	50 patients	Prospective Interventional Randomized Comparative Trial	Intralesional PRP injections	Mitomycin-C plus Dexamethasone	Penile curvature, IIEF-5 score, PDQ, plaque size	Improvement in PDQ domains and IIEF scores; curvature and erectile dysfunction improved in both groups, but more in PRP for erectile function; plaque size reduction noted in both groups	Small sample size, short follow-up duration, no placebo arm or blinding
**Virag, Sussman (** **21** **)**	2016	50 patients	Interventional Series	Intralesional injections of PRP+HA under US guidance	None	PDQ, IIEF-5, angulation, maximum thickness, patient satisfaction	38% reduction in average angulation, maximum thickness decreased from 4.4mm to 3.3mm, average PDQ bother reduced from 10.5 to 5, IIEF-5 increased from 17.7 to 21.1. 84% of patients showed improvement	No control group, short follow-up period, industryfunded
**Virag, Sussman (** **22** **)**	2016	75 patients	Case Control Study	PRP+HA injections under US guidance	None	Angulation, albuginea thickness, sexual activity, ED, PDQ	36.9% average angulation decrease, 26.7% reduction in albuginea thickness, improvement in erections in 37% of ED patients, 82.7% self-reported improvement, better results in non-calcified and <60° angulation cases	No control group for comparison
**Schirmann et al. (** **17** **)**	2022	17 patients	Prospective Pilot Study	Intra-plate injections of PRP	None	PDQ, Angle of curvature, Erectile function (IIEF-EF, EHS, SEP)	No side effects; PDQ domains significantly improved; curvature decreased by 11.8°; IIEF-EF score improved by 5-7 points	Small sample size, lack of control group, short-term study
**Alshuaibi et al. (** **23** **)**	2023	36 patients	Prospective Case Series Study	Combination of PNT, PM, and PRP injections	None	Improvement in curvature	Mean curvature improved by 16.85° (47.7% improvement); no serious events reported; effective for penile deformity due to PD	No control group, shortterm followup

Ongoing clinical trials are further investigating PRP's therapeutic role in PD. Early findings from Chu et al. indicate no adverse events, highlighting its safety. However, these initial results, based on a small cohort, showed no significant improvement in penile curvature at the 3-month evaluation. The study's authors note the need for further research to draw more definitive conclusions (24). Furthermore, long-term follow-up studies are necessary to assess the durability of PRP treatment effects in PD.

While short-term results are promising, the chronic nature of PD necessitates examination of long-term outcomes to fully understand the efficacy and safety of PRP therapy in this context. The American Urological Association (AUA) and European Association of Urology (EAU) guidelines acknowledge the current gaps in understanding the physiological impact of PRP therapy in PD and should currently be considered as being experimental (1, 25). This underscores the necessity for more comprehensive research to validate PRP as a viable treatment option for PD.

### Stem Cell Therapy (SCT)

The promise of restorative medicine, with a special emphasis on stem cells, lies in the fact that the ultimate measure of success, as defined by patients, is the achievement of a "cure" (27). Stem cells possess remarkable regenerative capabilities, primarily driven by their pleiotropic and paracrine effects (28). At present, mesenchymal stem cells (MSCs) represent the most widely used and accessible source of stem cells (29). Unlike embryonic derived stem cells, MSCs exhibit minimal tumorigenic potential and are not encumbered by ethical constraints (30). Initially characterized as a cell population with fibroblast-like properties originating from bone marrow (31), MSCs have subsequently been identified in various tissues, including muscle, brain, fallopian tubes, ligaments, synovium, and adipose tissue (32). Numerous preclinical in vitro and in vivo studies have demonstrated that these cells stimulate cell growth (via trophic effects), enhance cell survival and proliferation, facilitate neo-vascularization, promote re-epithelialization, and exert immunomodulatory effects by releasing a diverse array of cytokines (33).

Several studies have scrutinized the applicability of stem cells in rat models to address PD (34–37). These studies, collectively indicating improved erectile function and a reduction in PD associated alterations among rats subjected to stem cell treatment, highlight the potential benefits of this approach. In terms of ensuring the safety of stem cell administration, researchers have explored various routes, with the most prevalent approaches being intra scar-tissue or intracavernosal injection (38).

In 2013, a study used adipose tissue-derived stem cells (ADSCs) to treat PD in a rat model. Fibrosis was induced using TGF-β1 in the rat tunica albuginea (TA), followed by xenogeneic transplantation of human ADSCs within a day, resulting in notable improvements in penile fibrosis. This breakthrough marked the first successful instance of xenogeneic cell transplantation in immunocompetent animals without the need for immunosuppressants. ADSCs have demonstrated immunomodulatory and immunosuppressive properties in earlier research, including their effectiveness in reversing PD progression during the acute phase of TGF-β-induced inflammation and decreasing expression of tissue inhibitors of metalloproteinases. (35). In a related study simulating the chronic phase of PD, ADSCs were injected a month after TGF-β1 injection in a rat model. Remarkably, the rats exhibited reduced fibrosis, decreased collagen III expression, and lowered expression of fibrosis-related genes, indicating positive changes in biochemical fibrosis. Additionally, fibrotic plaques showed spontaneous partial regression after 60 days (34).

Another study was conducted utilizing stem cells to assess the potential of local autologous injection of the stromal vascular fraction (SVF) of adipose tissue in reducing established fibrosis in a rat model of PD. While no significant differences in erectile function were observed, there was a noticeable reversal of fibrotic changes after the SVF injection, highlighting the potential of local SVF injection to reverse TA fibrosis in the chronic phase of PD in a rat model (37).

Human studies to evaluate the feasibility of stem cell therapy for PD are scarce. Levy et al. published a compelling human study examining five patients with PD and penile deformities/curvatures ranging from 0º to 120º. The study involved intra-plate injections of placental matrix-derived stem cells (PM-MSC) to address this condition. Besides providing a notable improvement in curvature (by 30º to 120º) and reduction of the number of plaques, no complications involving penile hematoma, corporal rupture or penile edema occurred (7). This research marked the first instance of utilizing PM-MSC to manage PD in humans, albeit with a limited sample of five subjects. Another study, combining autologous SVF injections isolated from lipoaspirate with a series of ESWT, evaluated subjective outcomes and safety of this combined therapy in 11 men with stable PD. After a 6 months follow-up, all patients noted subjective improvement in curvature and subjective reduction in plaque size (38). The characteristics of the most relevant SCT studies are presented in Table-2.

**Table 2:** Studies examining the effects of SCT on PD.

STUDY	YEAR	STEM CELLS	HUMANS OR ANIMALS	RESULTS
**Castiglione et al. (** **34** **)**	2013	Humans adipose-derived stem cells	Animals	Erectile dysfunction improving during the acute phase of PD
**Gokce et al. (** **35** **)**	2014	Rat adipose-derived stem cells	Animals	Erectile dysfunction improving during the acute phase of PD
**Gokce et al. (** **36** **)**	2015	Genetically modified adipose tissuederived stem cells with human alfa-2b	Animals	Erectile dysfunction improving during acute phase of PD
**Milenkovic et al. (** **33** **)**	2019	Humans adipose-derived stem cells	Animals	Tunica albuginea fibrosis decreased in a rat model of chronic PD
**Hakim et al. (** **37** **)**	2020	Rat adipose-derived stem cells	Animals	Local injection of SVF in a rat model of chronic PD significantly decreased collagen III concentration in the TA
**Levy et al. (** **7** **)**	2015	Placental matrix-derived mesenchymal stem cells	Humans	Peak systolic velocity and penile curvature improved signifcantly 6 weeks, 3 months and 6 months after treatment. 7 of 10 fibrotic plaques in the tunica albuginea disappeared completely at 3 months

The cost of the off-label treatment expenses exhibited considerable variability among different clinics, with an average expenditure of $5,291 per stem cell therapy injection in USA (39). The AUA guideline regards the use of stem cells as a promising approach; however, it has not yet incorporated this treatment modality into its recommendations (1). The EAU does not mention the use of stem cells in its guideline on penile curvature.

### Extracorporeal shockwave therapy (ESWT)

The precise way in which ESWT impacts PD remains uncertain, despite numerous studies reporting positive outcomes. ESWT could potentially induce changes and restructuring in the penile plaque. Specifically, the application of ESWT might generate heat, leading to heightened local blood circulation (40). This, in turn, could trigger an inflammatory response, subsequently boosting macrophage activity. This cascade of events could eventually lead to the breakdown and absorption of the plaque (41).

Three studies (19, 42, 43) encompassing 225 patients were examined to gauge penile plaque size using ultrasonography. The results were compelling, showcasing a notable reduction in plaque size within the ESWT group when compared to the control group. Specifically, 39.8% of patients in the ESWT group experienced a reduction in plaque size compared to 30.3% observed in the control group. When it came to evaluating the improvement in penile curvature, researchers analyzed pre- and post-treatment photographs from three studies. According to the authors, 44% (37 of 84) of patients in the ESWT group reporting a significant improvement in penile curvature, slightly surpassing the 42.1% (48 of 114) noted in the control group. Additionally, the ESWT group demonstrated superior pain management, as 82.1% experienced pain relief and 61% achieved complete pain remission, surpassing the rates in the control group (51.6% for relief and 18.8% for complete remission).

In a study conducted by Di Mauro et al. analyzing 325 consecutive patients with PD in a multi-center single-arm clinical trial, notable improvements were observed. These improvements included a reduction in plaque size from 1.78 to 1.53 cm^2^, an increase in erect penis length from 13 to 14 cm, a decrease in penile curvature from 30.4 to 25.0 degrees, and a reduction in reported pain on the Visual Analog Scale (VAS) from 7 to 3. Furthermore, improvements in discomfort caused by PD, as indicated by the Peyronie's Disease Questionnaire (PDQ), and enhanced sexual satisfaction measured by the International Index of Erectile Function (IIEF), were also noted (8).

In 2021, Backr et al. conducted a comprehensive meta-analysis that revealed notable heterogeneity in the outcomes of individuals with PD undergoing ESWT. A total of three randomized clinical trials, comprising of 117 men in the ESWT group and 121 in placebo group were reviewed. Their analysis suggests that ESWT does not yield significant improvements in penile curvature or pain among men with PD. Nevertheless, there is evidence to suggest that ESWT may have a potential positive impact on reducing plaque size in this specific patient population (44). Table-3 shows relevant studies examining the effects of ESWT on PD (8, 42–46).

**Table 3:** Studies examining the effects of ESWT on PD.

AUTHOR	YEAR	STUDY TYPE	RESULTS
**Palmieri et al. (** **43** **)**	2009	Placebo- controlled randomized	ESWT treatment brought significant improvement in VAS, IIEF-5 and mean QoL scores. Mean plaque size and curvature were unchanged. ESWT leads to pain resolution and positively impacts erectile function and quality of life
**Hatzichristodoulou et al. (** **42** **)**	2013	Placebo controlled, randomized trial	ESWT is not recommend given the following: ESWT provide pain reduction by 85%. Pain resolution occurred in the placebo group by 48%. ESWT group showed no difference in plaque size reduction. Penile deviation worsened by 40% in ESWT group.
**Gao et al. (** **46** **)**	2016	Meta-Analysis	ESWT significantly increased the percentages in the following: lessening of penile plaques, pain relief and complete pain remission. There was insignificant improvement with penile curvature and sexual function in ESWT vs placebo.
**Di Mauro et al. (** **8** **)**	2019	Single-Arm Observational Study	ESWT treatment resulted in reduction in plaque size, penile curvature and pain. Penile lenght with erection increased after ESWT treatment
**Bakr et al. (** **44** **)**	2021	Systematic Review and Meta-Analysis	ESWT is associated with a reduction in plaque size but no significant difference in penile curvature, erection function or pain
**Abdessater et al. (** **45** **)**	2022	Retrospective Data Analysis	ESWT had positive impacts on penile pain, curvature, plaque size and erectile dysfunction in PD during the early inflammatory phase

According to the guidelines of the AUA, clinicians are advised to refrain from using ESWT for the purpose of reducing penile curvature or plaque size. However, healthcare professionals may consider the possibility of offering ESWT to alleviate penile pain. This is a conditional recommendation with an evidence strength grade of B (1). As per the EAU, ESWT may be offered only to treat penile pain in the acute phase of PD, with a level of evidence 2b. (26). Patients need vigilant monitoring for occurrences of localized pain, hematoma formation, neurapraxia, and other adverse events, despite complications not commonly manifesting (46). Further research is warranted to unravel ESWT's full potential and optimize its application in treating PD.

## CONCLUSIONS

Restorative therapies have emerged as an innovative and less invasive treatment option for PD. Results from current studies appear to be promising and demonstrate good safety profile. However, at the moment, these treatments do not provide cure for men diagnosed with acute or chronic PD. Unfortunately, due to scarce evidence, PRP and SCT are still considered by AUA and EAU guidelines as experimental therapies. ESWT is recommended, by the same guidelines, for pain management. More high-quality studies with long-term follow-up outcomes are needed to evaluate efficacy, reproducibility and define evidenced-based protocols to standardize techniques.
